# Delivery of nucleic acids using nanomaterials

**DOI:** 10.1186/s43556-023-00160-0

**Published:** 2023-12-14

**Authors:** Yuyang Qin, Liyuan Ou, Lili Zha, Yue Zeng, Ling Li

**Affiliations:** West China School of Public Health and West China Fourth Hospital, and State Key Laboratory of Biotherapy, Sichuan University, Chengdu, 610041 China

**Keywords:** Organ-selective delivery, Nucleic acids, Nanomaterials, Non-viral vectors

## Abstract

The increasing number of approved nucleic acid therapeutics demonstrates the potential for the prevention and treatment of a broad spectrum of diseases. This trend underscores the significant impact and promise of nucleic acid-based treatments in the field of medicine. Nevertheless, employing nucleic acids as therapeutics is challenging due to their susceptibility to degradation by nucleases and their unfavorable physicochemical characteristics that hinder delivery into cells. Appropriate vectors play a pivotal role in improving nucleic acid stability and delivering nucleic acids into specific cells. The maturation of delivery systems has led to breakthroughs in the development of therapeutics based on nucleic acids such as DNA, siRNA, and mRNA. Non-viral vectors have gained prominence among the myriad of nanomaterials due to low immunogenicity, ease of manufacturing, and simplicity of cost-effective, large-scale production. Here, we provide an overview of the recent advancements in nanomaterials for nucleic acid delivery. Specifically, we give a detailed introduction to the characteristics of polymers, lipids, and polymer-lipid hybrids, and provide comprehensive descriptions of their applications in nucleic acid delivery. Also, biological barriers, administration routes, and strategies for organ-selective delivery of nucleic acids are discussed. In summary, this review offers insights into the rational design of next-generation delivery vectors for nucleic acid delivery.

## Introduction

**Fig. 1:**
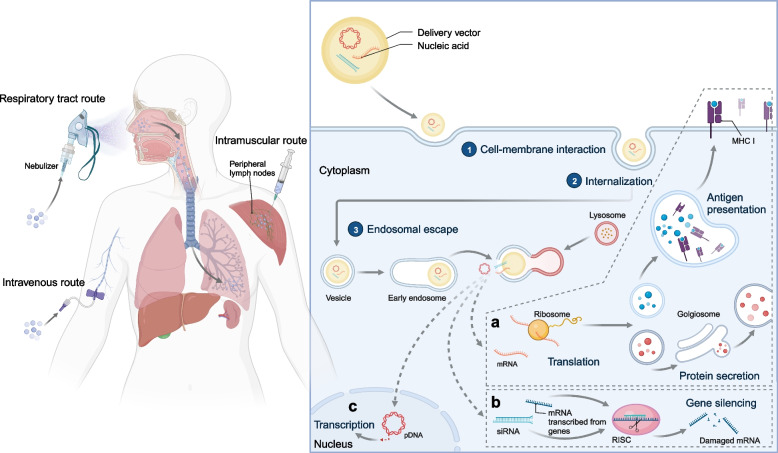
Schematic illustration depicting nanoparticles' route and functional mechanisms within cells. When nanoparticles contact cells, they are recognized and internalized by the cell membrane. Inside the cell, the lysosome-endosome system engulfs the nanoparticles, where the cargo they carry is subsequently released. **a** mRNA is translated by the ribosome in the cytoplasm, and the resulting protein may be processed by the golgiosome for secretion or presented as an antigen. **b** siRNA binds with the RNA-Induced Silencing Complex (RISC) in the cytoplasm, inhibiting the translation of mRNA transcribed from genes. **c** pDNA crosses the nuclear membrane and undergoes transcription in the cell nucleus. Created with BioRender.com

Nucleic acid therapy offers a novel therapeutic modality for congenital and acquired diseases by delivering exogenous nucleic acids into lesions to modulate the expression of proteins [1–5] (Fig. 1). Compared to conventional small-molecule and antibody drugs, nucleic acid drugs have advantages of short development cycle, abundant choice of targets and remarkable curative effect [6–10]. Furthermore, nucleic acid drugs offer a solution to circumvent the limitations associated with undruggable targets [11–13]. These advancements provide potential treatment alternatives for previously considered incurable conditions [6, 14]. With the rapid development of molecular biology techniques such as in vitro transcription (IVT) and Clustered regularly interspaced short palindromic repeats associated (CRISPR-Cas) gene editing, nucleic acid drugs have made tremendous advances in the prevention and treatment of a variety of diseases [15–19].

Naked nucleic acids face numerous challenges in in vivo delivery [20]. The key to achieving the complete functions of nucleic acid drugs is the choice of delivery vectors [21]. Ideally, these vectors should protect nucleic acids from enzymatic degradation, facilitate cellular uptake and endosomal escape, and exhibit minimal toxicity. Viral vectors, such as adenovirus, lentivirus, and adeno-associated virus, have been developed to enable efficient cellular uptake [22–24]. Several nucleic acid drugs delivered using viral vector platforms, such as Luxturna® and Zolgensma®, have received approval from the U.S. Food and Drug Administration (FDA) [25]. Generally, viral vector-based nucleic acid drugs have long-lasting efficacy over conventional medications, only a single injection of these nucleic acid drugs maintains therapeutic efficacy for a considerable length of time [22, 26–29]. Nevertheless, viral vectors can potentially induce antiviral immune responses, which poses a challenge for repeat administration [26, 29, 30]. In addition, adverse reactions of abnormal blood clots occurred in some instances of clinical use of viral vector nucleic acid drugs [29–31].

There has been a growing interest in structuring non-viral vectors in recent years. Compared with viral vectors, non-viral vectors exhibit low immunogenicity without the risk of insertional mutagenesis and ease of large-scale production [32, 33]. Conventional non-viral vectors generally suffer from low transfection efficiency and limited tissue targeting [34]. Fortunately, novel nanomaterials with superior cell transfection and active tissue targeting capability have emerged [35, 36]. For instance, lipid nanoparticles (LNPs) have shown significant potential in nucleic acid drug delivery, as evidenced by numerous preclinical and clinical studies [37, 38]. One notable example is the siRNA-LNP drug Onpattro® (patisiran), which has gained positive outcomes in treating Hereditary Transthyretin-Mediated (hATTR) amyloidosis with only one dose every three weeks and received FDA’s approval in 2018 [39–42]. Since Onpattro's approval, five siRNA-based drugs have been available on the market, and over 200 siRNA therapies are currently in development.

Most recently, the global SARS-CoV-2 pandemic has triggered an unprecedented emergence of mRNA-based vaccines for infectious diseases. Among these candidates, BNT162b2 (Comirnaty®) by BioNTech/Pfizer and mRNA-1273 (Spikevax®) by Moderna have demonstrated high effectiveness in the prevention and control of SARS-CoV-2 [43, 44]. mRNA vaccines have great promise in terms of preventing and treating pandemics owing to high efficacy, ease of manufacture, scalable production, and high success rate of clinical trials [45–47]. In March 2023, SYS6006, an mRNA vaccine developed by the CSPC Pharmaceuticals Group, became China's first domestic COVID-19 mRNA vaccine for emergency use. A recent survey identified 966 vaccine candidates in development worldwide, of which approximately 20% are nucleic acid-based vaccines [48]. They have the potential to usher in a new era of pandemic prevention and control, opening up new opportunities for drug discovery and development [46, 49–53]. So far, nucleic acid drugs have remarkably progressed in treating liver, eye, and cardiovascular diseases [54–60].

In this review, we focus on recent advances in nanomaterials, including polymers, lipids, and polymer-lipid hybrids, as vehicles for nucleic acid delivery. The physiological barriers and the diverse administration routes are also described. Understanding these factors is crucial for rationally designing optimal vectors for targeted delivery of nucleic acids to ensure both efficacy and safety. Overall, we aim to offer insights into developing next-generation delivery carriers for nucleic acids with optimal properties.

## Biological barriers for nucleic acid delivery

### Systemic barriers

**Fig. 2:**
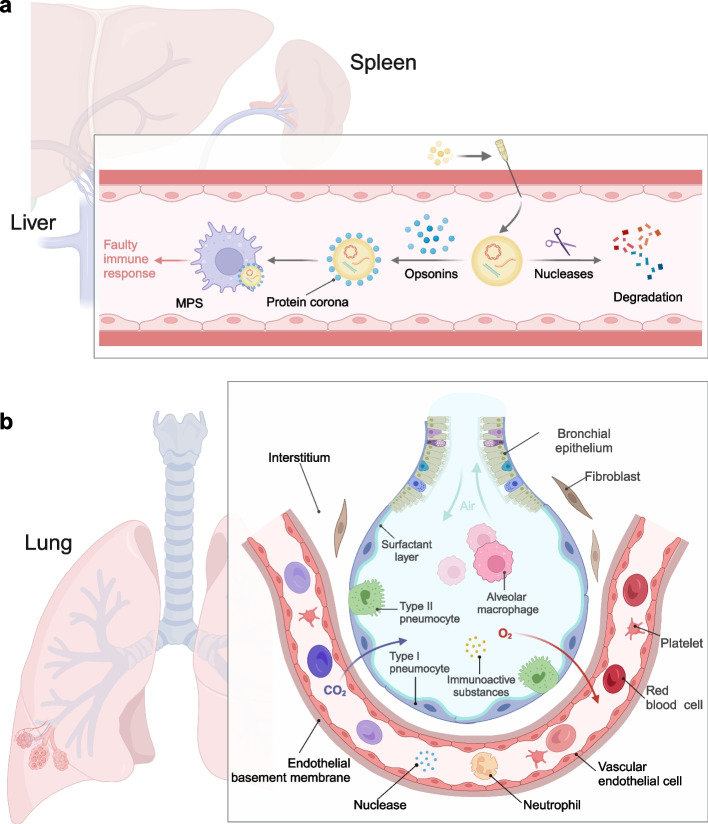
**a** Effect of factors such as blood nuclease and MPS on nanoparticles. **b** Alveolar-capillary physiology and barrier effect on respiratory delivery of drugs. Created with BioRender.com

Before reaching target cells, nanoparticles face a barrier known as the extracellular barrier, which exists within the bloodstream and intercellular matrix (Fig. 2a). This barrier primarily includes the complex components in the blood. The blood serum contains a large number of nucleases, including endonucleases and exonucleases, which can hydrolyze the phosphodiester bonds of exposed nucleic acids and inactivate them [61]. In early research concerning nucleic acid delivery, nucleic acids were tightly bound to carrier materials or chemically modified to prevent nuclease-induced degradation. However, these strategies have several drawbacks compared to the utilization of advanced delivery carriers [62]. Like all foreign substances, once nanoparticles are recognized by the mononuclear phagocyte system (MPS), they often undergo degradation and trigger undesired immune reactions in inappropriate sites. After entering the bloodstream, the surface of nanoparticles quickly adsorbs a layer of proteins, including opsonins, serum albumin, complement, and others, referred to as the “protein corona” [63–65]. The presence of the protein corona not only enhances the phagocytosis of nanoparticles by phagocytic cells [65] but may also mask ligands on the nanoparticle surface responsible for active targeting [66], leading to a decrease in nanoparticle specificity. It has been found that polyethylene glycol (PEG) modification prevents protein corona formation and reduces MPS clearance [67]. Similarly, Tasciotti et al. developed a coating of bionic particles consisting of cell membranes isolated from leukocytes, reducing MPS's conditioning effect [68].

### Organ barriers

Nanoparticles targeting organs other than the liver and kidneys also face the risk of hepatic and renal clearance, mainly related to the nanoparticles' properties (size, surface charge, etc.). Nanoparticles with a diameter of less than 5 nm are rapidly excreted through the kidneys after entering the bloodstream. In contrast, larger nanoparticles are eliminated by the liver's reticuloendothelial system (RES) [69, 70]. In addition, particle size is related to the passage of nanoparticles to tissue-specific structures. For example, nanoparticles targeting the spleen must also overcome the splenic barrier after spilling out of the blood vessels. Macrophages in the red medulla and marginal zone of the spleen prevent the nanoparticles from entering the white medulla, thus lessening the strength of the immune response [69]. Only nanoparticles with appropriate size can cross the marginal zone through the bridging channel and enter the T-cell and B-cell zones in the white medulla, which are densely populated with lymphocytes that can quickly and efficiently initiate an adaptive immune response against specific antigens. Moreover, the human body houses various organs, such as the brain and placenta, which possess robust protective mechanisms. Additional obstacles must be overcome to successfully transport nanoparticles to these organs, such as the blood–brain barrier for delivering nucleic acids to the brain and the blood-labyrinth barrier for targeting the inner ear [71–73].

The case of the lungs is even more specific, where the delivery of nanoparticles to the lungs via the respiratory tract, such as inhalation, must overcome a complex respiratory barrier (Fig. 2b). The mucus-cilia clearance system (MC) on the airway epithelium is an initial barrier to overcome [74]. Airway mucus, derived mainly from submucosal glands and goblet cells, consists of mucin fibers forming a dense meshwork [75]. The mucus layer immobilizes inhaled foreign bodies, including nucleic acid-nanocarrier complexes, through electrostatic interactions and removes them from binding sites. Studies have shown that only particles smaller than 40 nm can effectively penetrate the mucus layer through passive Brownian motion [76]. In diseases like cystic fibrosis and asthma, excessive mucus accumulation hinders drug delivery and induces coughing, which further clears foreign substances [77]. Researchers are attempting to overcome the barriers of mucociliary clearance by utilizing material properties [78, 79]. Angelo and others have developed mucus-inert nanomaterials that exploit the intrinsic properties of the mucus layer to enable sustained siRNA release within the mucus layer [80]. Additionally, due to proteins, lipids, and ions in the airways, cationic polymer- and lipid-based carriers interact readily with negatively charged mucus components and aggregate [81]. Surfactant, a lipoprotein secreted by alveolar type II epithelial cells, is another barrier to the pulmonary delivery of nucleic acids. It comprises dipalmitoyl phosphatidylcholine (DPPC) and surfactant-binding protein (SP) and significantly affects the bio-efficacy of RNA-nanoparticle complexes [82]. In animal experiments, adding the surfactant “Alveofact” reduced the transfer efficiency of DNA-PEI complexes [83].

### Cellular barriers

Once nanoparticles successfully circumvent the barriers mentioned above and approach target cells, the cell membrane becomes the first obstacle encountered during cellular uptake. Unlike small molecule drugs, nanoparticles rely on active uptake mechanisms to enter cells, including receptor-mediated endocytosis, macropinocytosis, and caveolae-mediated endocytosis [84]. Overall, cellular uptake of nanoparticles can be categorized into two types: phagocytosis and pinocytosis [84, 85]. Phagocytosis only occurs in phagocytic cells such as monocytes, neutrophils, and macrophages, while pinocytosis is widely present in various cell types. The cellular uptake of nanoparticles is typically receptor-mediated, where ligands on the nanoparticles bind to specific receptors on the cell membrane, leading to membrane invagination. Different nanoparticles employ distinct pathways to breach the cell membrane. Kubota et al. treated HeLa cells with specific inhibitors targeting different uptake pathways and found that cellular uptake of lipid nanoparticles (LNPs) mainly relies on caveolae-mediated endocytosis, while the uptake of lipoplexes depends on at least two pathways [86]. Efficient cellular uptake is a prerequisite for enhancing the cell availability of nanocomplexes carrying nucleic acids. The composition, size, surface charge, and shape of nanoparticles can influence cellular uptake efficiency [86–88]. Notably, the protein corona is not the sole factor affecting the interaction between nanoparticles and target cells. Suberi et al. found that the content of PEG on the surface of polymers influences their cellular uptake efficiency. However, this influence is not linear. In-depth studies have revealed that high PEG density can affect the peripheral conformation of polymers, impacting the interaction between nucleic acid-loaded nanoparticles and target cells [89]. Hatakeyama et al. also reached a similar conclusion when using a different material to deliver siRNA [90].

The vesicles formed during the endocytosis mentioned above fuse with early endosomes within the cell and gradually mature into late endosomes through acidification, ultimately combining with lysosomes. Lysosomes are acidic organelles within the cell with a firm acidity (pH around 5 [91, 92]) and contain various enzymes. Therefore, if the nanocarriers loaded with nucleic acids cannot escape promptly, both the carrier and its cargo will be destroyed in the extreme environment of the lysosome. Different materials used to form nanoparticles have different mechanisms for endosomal escape. However, most nanoparticles, including cationic lipid nanoparticles, disrupt the negatively charged endosomal membrane through electrostatic interactions [93, 94]. For example, it is widely believed that some cationic polymers interact with the endosomal membrane through the “proton sponge effect” and subsequently disrupt it (which will be described in detail in the corresponding section) [95]. This interaction can also be achieved using ionizable lipids, as these pH-sensitive lipids can be protonated in acidic environments, acquiring a positive charge [96]. It is worth noting that some helper lipids can also be protonated in acidic environments. For example, 1,2-dioleoyl-sn-glycero-3-phosphoethanolamine (DOPE) with a hexagonal inverted cone structure can form ion pairs with membrane phospholipids in the endosome, thereby promoting the process of endosomal escape [97]. Another promising approach is to conjugate viral-derived proteins or peptides on the surface of nanoparticles, utilizing the natural entry mechanisms of viruses to achieve endosomal escape. For example, the HIV-1 transmembrane protein gp41 and H5WYG are derived from the influenza virus [98–100].

## Delivery routes for nucleic acids

### Systemic routes

**Fig. 3:**
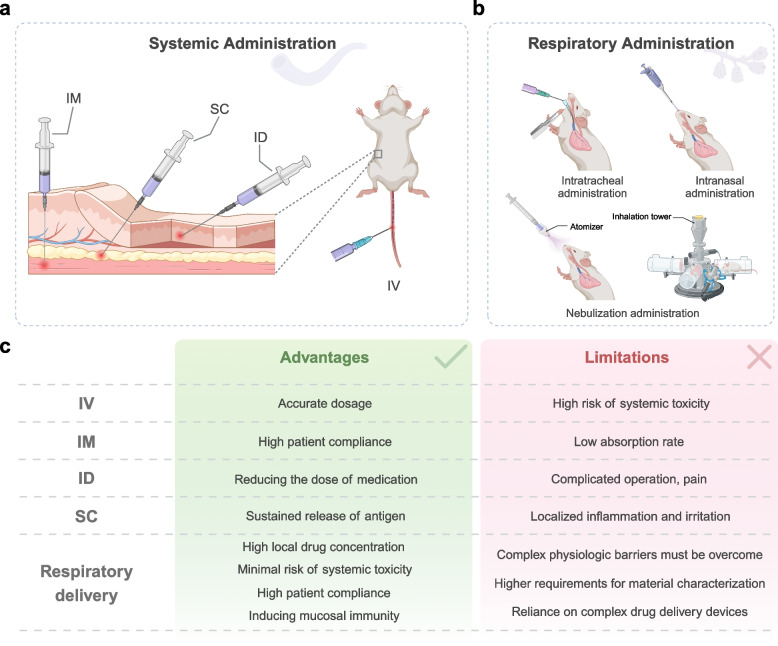
**a**, **b** Illustration of the main routes of administration of nucleic acid drugs or vaccines. **c** Comparison of the advantages and limitations of different delivery routes of nucleic acid drugs or vaccines. Created with BioRender.com

Systemic administration mainly includes subcutaneous injection (SC), intramuscular injection (IM), intradermal injection (ID), and intravenous injection (IV) (Fig. 3). Among them, the nanoparticles administered via SC, IM, and ID are not directly entering the bloodstream but are taken up by APCs in the subcutaneous or muscle tissue [101]. Each of these injection methods has its pros and cons. For example, it is generally believed that subcutaneous injection has a lower capillary density, allowing the antigen to be continuously released at a certain rate after SC [102]. Still, some argue that SC is an outdated immunization method [103]. Although nucleic acid drugs or vaccines administered through SC, IM, and ID mainly activate immune cells in peripheral lymph nodes, it is difficult to achieve targeted delivery to central immune organs or other specific tissues to date. Owing to their higher safety and patient compliance compared to IV, these routes remain the mainstay of preventive vaccination. Generally, most nanoparticles targeting a specific tissue efficiently rely on the IV route. In this case, nanoparticles enter the bloodstream at the fastest rate after injection and participate in blood circulation. Nucleic acid-nanocarrier complexes tend to passively target tissues with abundant blood supply, especially for nucleic acid drugs targeting the liver, IV is an excellent delivery route.

Despite the advancements in nucleic acid delivery technologies using various materials, achieving efficient delivery of nucleic acid-nanocarrier complexes via systemic administration to extrahepatic tissues remains challenging [104–106]. When nucleic acid-nanocarrier complexes are injected intravenously, due to the dependence on the circulatory system for drug transport from the injection site to the target cells, a large amount of the complex is intercepted mainly by hepatocytes, resulting in a significant release of nucleic acids in the liver, and consequently, a lower-than-anticipated concentration of the drug reaching the target tissues. Researchers have thought of many ways to achieve extrahepatic targeting of nucleic acids. In the case of LNPs-RNA, for example, the scientific community has been exploring three main directions to achieve extrahepatic targeting. These approaches aim to enhance the targeted delivery of nucleic acid-nanocarrier complexes to the target tissues and improve their therapeutic efficacy while overcoming the challenges associated with systemic administration.

1. Pre-injecting a large number of irrelevant liposomes (Nanoprimers) to occupy hepatocyte sites and slightly weaken the uptake of LNPs by hepatocytes [107]. Regardless, the clinical translational potential of this approach is limited.
2. Modifying ligands by splicing to improve the existing LNPs. For example, Lee et al. enhanced the delivery and transfection efficiency of siRNA in cancer cells by conjugating the EphA2 ligand, Ephrin-A1, on the surface of LNPs, taking advantage of the overexpression of the EphA2 receptor in most cancer cells [108].
3. Designing and synthesizing new LNPs with endogenous targeting functions. Qiu et al. discovered that LNPs containing an amide bond in their lipid tails selectively delivered mRNA to mouse lung cells, especially alveolar endothelial cells. They also altered the selectivity of drug delivery to alveolar endothelial cells or alveolar macrophages by adjusting the structure of the head of lipids [109]. Moreover, changing the components of LNPs can achieve selective targeting of tissues and organs.

### Respiratory routes

Given the challenges of systemic drug delivery, topical delivery techniques have received increasing attention in recent years [110]. Theoretically, the respiratory route is optimal for lung epithelial cells targeting [111]. This approach has successfully prevented and treated viral infections and respiratory diseases [112–115]. Unlike other organs, the lungs are directly exposed to the external environment through the respiratory tract, and the mucosa of the respiratory system provides a vast absorptive surface area. This unique characteristic makes the delivery of nucleic acid-nanocarrier complexes to the lungs through intratracheal/intranasal drops and nebulization a highly promising strategy [116–118]. By delivering the complexes via the respiratory tract, complexes can directly reach cells, such as alveolar epithelial cells, in the pulmonary airways and alveoli without relying on systemic circulation. This approach achieves efficient drug delivery and minimizes drug loss [119].

Inhalation presents great potential among various routes for delivering nucleic acid-nanocarrier complexes to the respiratory tract [120]. This route typically requires the use of specialized devices. Early equipment involved metered-dose inhalers (MDIs), which deliver drugs dissolved or suspended in a liquid propellant (e.g., hydrofluoroalkanes) to achieve rapid and convenient drug delivery. Later, dry powder inhalers (DPIs) were introduced to overcome the problem of limited drug deposition in the lower respiratory tract by MDIs, which deliver drugs in the form of solid aerosols. Nevertheless, the efficiency of dry powder inhalers is mainly dependent on the patient's respiratory function, posing new challenges for quality control stability [121]. Subsequently, nebulization devices were developed. Dolovich et al. demonstrated that the liquid aerosols produced by nebulizers, capable of carrying hundreds of nanoparticles per drop, can reach nearly all regions of the lungs [122]. Philip J. Santangelo [123, 124] and Daniel G. Anderson's team [114] achieved effective deposition and efficient transfection of functional mRNA in the lungs of experimental animals using devices such as small animal vibrating sieve mesh nebulizers and nebulizing towers, demonstrating the role of nebulization in delivering nucleic acids to counter respiratory pathogens. It's worth mentioning that ALN-RSV01, a nebulized siRNA therapy targeting the RSV nucleocapsid protein (N), has been clinically validated and proven safe and effective in over 3,000 symptomatic patients [125]. Despite that, nebulized drug delivery also faces many challenges due to the instability of liquid nucleic acid-nanocarrier complexes. Nebulization devices like jet and ultrasonic nebulizers may apply a continuous shear force to the complexes, leading to nanoparticle coalescence. Consequently, nebulization technology poses challenges related to delivery materials, buffer systems, and nebulization device design [126].

## Organ-selective nucleic acid delivery

Due to the unique physiological configuration and function of the liver, nucleic acids carried by nanomaterials such as LNPs tend to aggregate in the liver [127], granting nucleic acids unique advantages in treating liver diseases. So far, among the five siRNA drugs approved by the FDA, except for Onpattro®, all have employed GalNAc-based modifications. This proven strategy allows nucleic acid drugs to achieve efficient liver parenchymal cell targeting [128, 129]. However, the liver has a complex cellular composition. In addition to hepatic parenchymal cells, the hepatic microenvironment includes hepatic stellate cells (HSCs), Kupffer cells (hepatic macrophages), and hepatic endothelial cells [130], which have been associated with a wide range of liver diseases and metabolic disorders. For example, HSCs are considered significant cells in the formation of hepatic fibrosis and are closely associated with various chronic liver diseases [131]. It is necessary to develop better strategies targeting multiple cell types.

Efficiently modulating the immune system is crucial for treating diseases such as infectious diseases, tumors, and autoimmune diseases. In this regard, nucleic acid drugs also have advantages. The liver is also a component of the body's immune system [132]. However, secondary lymphoid organs, including lymph nodes (LN) and spleen, are the core foundation of immune responses [133]. The spleen, the largest lymphoid organ, stores about one-third of circulating lymphocytes and possesses a large number of antigen-presenting cells (APCs) and tissue-resident lymphocytes [134]. After intravenous administration, nanocomplexes targeting the spleen can be internalized by the large number of APCs in the spleen, resulting in mighty and enduring cellular and humoral immunity [135]. Thus, the spleen has become another promising organ for nucleic acid delivery, which has greatly attracted a lot of interest.

Unlike other organs, the lungs are immediately exposed to the external environment through the respiratory tract, making them susceptible to a wide range of pathogens [136]. Recently, increasing environmental pollution and outbreaks of pandemics, such as Influenza, respiratory syncytial virus (RSV) infection and COVID-19 [137, 138], have led to a significant rise in the incidence of pulmonary diseases. It has imposed an enormous burden on society and even triggered global public health crises on multiple occasions [139, 140]. Nucleic acid drugs present remarkable strengths in the treatment of inherited lung diseases [141], lung cancer [142], asthma [143], and infectious pneumonia [144]. Over the past few years, several lung-targeted nucleic acid drugs, including MRT5005®, RCT1100®, VX-522®, and ARO-ENaC®, have entered clinical trials (Table 1). However, the majority of lung-targeted nucleic acid drugs remain in the preclinical stage, restricting breakthroughs in these studies due to the lack of appropriate delivery materials [145–148].

**Table 1:** Clinical trials based on siRNA or mRNA therapy for lung diseases

Drug name	Sponsor	Cargo	Disease	Administration route	Nanocarrier	Trial number	Phase	Status
MRT5005	Translate Bio	mRNA	Cystic Fibrosis (CF)	Inhalation (Nebulization)	LNP	NCT03375047	1/2	Unknown
RCT1100	ReCode	mRNA	Primary Ciliary Dyskinesia (PCD)	Inhalation (Nebulization)	LNP (SORT)	NCT05737485	1	Recruiting
VX-522	Vertex-Moderna	mRNA	Cystic Fibrosis (CF)	Oral inhalation (Nebulization)	LNP	NCT05668741	1	Recruiting
ARCT-032	Arcturus	mRNA	Cystic Fibrosis (CF)	Inhalation (Nebulization)	LNP (LUNAR®)	NCT05712538	1	Recruiting
ARO-ENaC	Arrowhead	siRNA	Cystic Fibrosis (CF)	Inhalation (Nebulization)	Chemical Modification (Targeting ligands)	NCT04375514	1	Terminated (Adverse reaction)
ARO-MUC5AC	Arrowhead	siRNA	Muco-obstructive, Chronic Obstructive Pulmonary Disease (COPD), etc	Inhalation (Nebulization)	Chemical Modification (Targeting ligands)	NCT05292950	1/2	Recruiting
ARO-RAGE	Arrowhead	siRNA	Inflammatory Lung Disease (e.g. asthma)	Inhalation (Nebulization)	Chemical Modification (Targeting ligands)	NCT05276570	1/2	Recruiting
ARO-MMP7	Arrowhead	siRNA	Idiopathic Pulmonary Fibrosis (IPF)	Inhalation (Nebulization)	Chemical Modification (Targeting ligands)	NCT05537025	1/2	Recruiting
TRK-250 (BNC-1021)	Toray-Bonac	siRNA	Idiopathic Pulmonary Fibrosis (IPF)	Inhalation	Chemical Modification (PnkRNA)	NCT03727802	1	Completed
NBF-006	Nitto	siRNA	Non-Small Cell Lung Cancer (NSCLC)	Intravenous infusion	LNP	NCT03819387	1	Recruiting

The clinical trials were from ClinicalTrials.gov

## Nanomaterials for nucleic acid delivery

To protect nucleic acids and ensure efficient transfection, encapsulating them into nanoparticles using safe materials is often necessary. However, selecting suitable materials presents a challenge, as they must achieve effective encapsulation, be taken up by target cells, and successfully deliver the gene to the intended site of action within the cell. Additionally, these materials should possess good biosafety and degradability properties. The success of organ-targeted nucleic acid delivery using nanomaterials should circumvent various extracellular barriers and intracellular barriers. Different routes of drug delivery are associated with distinct physiological barriers. In the case of respiratory tract delivery, the challenge lies in overcoming the barriers presented by the highly branched respiratory tract, characterized by varying diameters and lengths. Nanoparticles, due to their nanoscale dimensions, are quickly exhaled. Therefore, specific methods, such as integrating nanoparticles with excipients, need to be employed to confer inhalation characteristics upon the particles. Furthermore, respirable particles tend to accumulate in the mucus layer, preventing their entry into cells. When administered via intravenous injection, drugs must traverse the vessel wall and be endocytosed by cells, subsequently releasing the encapsulated nucleic acids through endosomes. Consequently, delivery carriers for nucleic acids should possess enhanced endosomal escape capabilities. A comprehensive understanding of diverse nanomaterials' chemical properties and applications is crucial in the rational development of more effective and selective nucleic acid delivery vehicles catering to treating specific tissues and cells.

### Polymer-based delivery system

#### Polyethyleneimine (PEI)

**Fig. 4:**
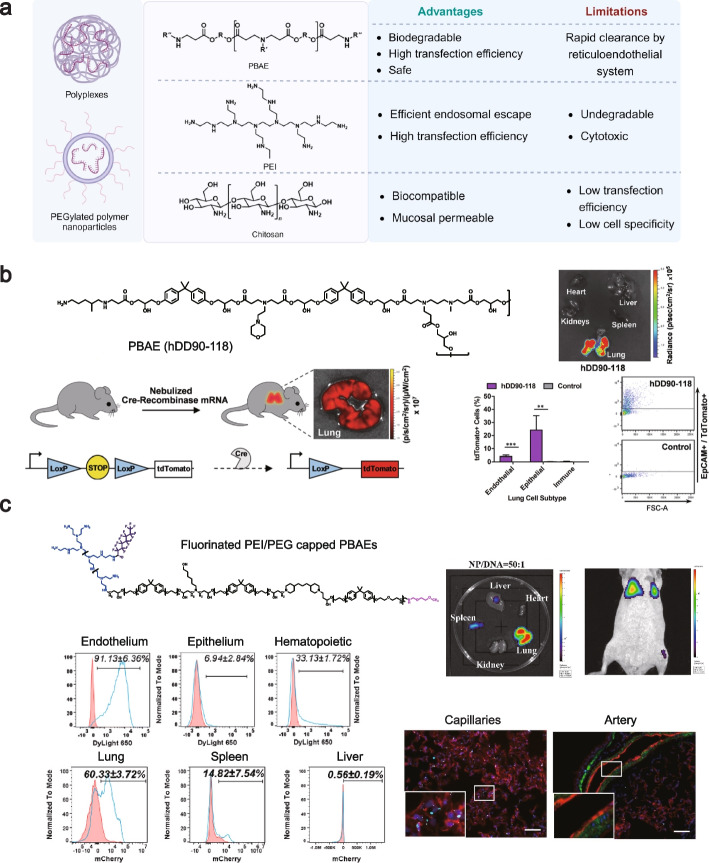
**a** Schematic illustration, chemical structures, and comparison of advantages and drawbacks of several polymers used as nucleic acids nanocarriers. Created with BioRender.com. **b** hDD90-118 was synthesized by adding N-methyl-1,3-diaminopropane, a tri-functional amine. Bioluminescence 24 h after inhalation of hDD90-118 polyplexes. hDD90-118 vectors produced significantly higher radiance localized to the lung. A Cre-loxP mouse model was utilized to quantitatively assess the lung cell subtypes transfected by hDD90-118 polyplexes. The tdTomato + lung cells were typed using flow cytometry, where endothelial cells were labeled with CD31, epithelial cells with EpCAM, and immune cells with CD45. Reprinted with permission [114]. Copyright 2019, Wiley. **c** The schematic of the PBAE used in the article. IVIS shows the biodistribution of luciferase expression in dissected mouse organs at a nanoparticle-to-DNA ratio of 50:1. IVIS whole-body imaging shows the in vivo bioluminescence following treatment with PBAE nanoparticles. Flow cytometry analysis of the PBAE nanoparticle distribution (blue curve) in different respiratory cell types compared to the control (red curve). Flow cytometry shows mCherry reporter activity in endothelial cells isolated from different murine organs. PBAE nanoparticles (blue curve); Untreated control (red curve). Immunofluorescence of frozen lung sections after I.V. injection of DyLight 650-labeled nanoparticles. The PBAE nanoparticles (light blue); Endothelial cells (CD31, red); Smooth muscle cells (αSMA, green); nucleus (DAPI, dark blue). Reprinted with permission [154]. Copyright 2023, KeAi

Polyethylenimine (PEI) is a highly charged cationic polymer that easily binds negatively charged nucleic acids to form complexes transfected into adherent and suspension cells, and is commonly used for transient gene transfer (Fig. 4a). Moreover, PEI is easily synthesized, allowing for flexible adjustment of its physicochemical properties. It demonstrates significant transfection efficiency in delivering nucleic acids in vivo. The main advantage of PEI as a nucleic acid delivery material lies in its efficient endosomal escape. The “proton sponge effect” is considered one of the critical principles. PEI can capture a large number of protons. Then, the influx of chloride ions and water into endosomes/lysosomes disrupts osmotic pressure homeostasis, leading to endosomal rupture and subsequent release of nucleic acids [95]. These characteristics have positioned PEI as extensively employed vectors for the delivery of various nucleic acids, including plasmid DNA and siRNA, to diverse tissues [149–151]. However, this disruptive effect is not limited to endosomal membranes, the uncontrollable proton sponge effect along with the high-density charge of the polymer, can potentially damage cell membranes, and mitochondria and even induce cell necrosis [152]. Moreover, the difficulty of PEI biodegradation and its cytotoxicity have limited its widespread application as a nucleic acid delivery material [153].

To address these limitations, efforts are being made to improve the safety of PEI while maintaining its delivery efficiency. Strategies such as reducing the molecular weight of PEI, decreasing branching structures (e.g., deacetylation), hydrophobic modification, or conjugation with other polymers (e.g., PEG, chitosan, hyaluronic acid) to form nanoparticles have shown promise [155, 156]. For example, using PEG-PEI as a siRNA delivery system may reduce cytotoxicity while maintaining the target gene silencing effect. It should be noted that PEGylated modifications have the potential to initiate immune responses and inflammatory responses in the lungs but are generally not sufficient to cause lung tissue damage [126, 157]. In another example, it was observed that the ^32^P-siRNA-PEI complex showed a minimal reduction in the radiolucent signal detected after treatment compared to the naked ^32^P-siRNA. Of note, the presence of PEI considerably influenced the uptake of ^32^P-siRNA by bronchoalveolar lavage cells (BAL cells) [158, 159].

Daniel J. Siegwart’s group found that fluorinated PEI successfully mediated siRNA binding in the triple-negative breast cancer (TNBC) cell line, MDA-MB-231, while reducing cytotoxicity and aggregation compared to unmodified PEI. Moreover, fluorinated PEI caused a distinct shift in the biodistribution of siRNA from the lungs to the liver [103]. Fernando et al. showed that pDNA was transfected into retinal pigment epithelium (ARPE-19) and human hepatocellular carcinoma (HepG2) cell lines via succinic acid-modified PEI. They found that adding succinic acid slightly reduced the strength of the polymer-DNA interaction, resulting in better intracellular DNA release and reduced cytotoxicity by avoiding the adsorption of proteins onto the polymers [160]. In a separate study, Kurosaki et al. observed that the positive charge of the pDNA/PEI complex was effectively shielded by the addition of 1,2-dioleoyl-sn-glycero-3-phospho-L-serine (DOPS). In their finding, the gene was selectively highly expressed in the spleen after intravenous injection of the pDNA/PEI/DOPS ternary complex [161]. Yang et al. designed a biocompatible biomimetic system based on nano-erythrocyte bodies (NER) and black phosphorus nanosheets (BP) to achieve spleen targeting. BP was covalently modified with PEI and served as the core to efficiently condense mRNA via electrostatic interactions, forming NER@BPmRNA. In in vivo experiments, they demonstrated that NER@BP, when injected into the muscle, effectively targeted the spleen for antigen delivery [162].

#### Chitosan

Chitosan is a naturally occurring and biocompatible polysaccharide material that exhibits positive electrical properties due to the presence of amine groups [163–165] (Fig. 4a). These positive charges enable chitosan to encapsulate nucleic acids effectively. In addition, chitosan possesses immunomodulatory properties and, therefore, has the potential to be used as an adjuvant for nucleic acid vaccines [166, 167]. The adhesive and mucosal permeable properties of chitosan have been reported to contribute to the delivery of nucleic acids to the lungs through the respiratory tract [105]. For example, Silva et al. showed that chitosan and siRNA powders prepared using carbon dioxide-assisted spraying with dry drying (SASD) technique effectively deposited in the mouse lungs after administration [168]. Okamoto et al. also used chitosan as a carrier to deliver pDNA as a dry powder. They found that chitosan as a dry powder resulted in higher expression of pDNA compared to intravenous or intratracheal infusion administration [169].

However, the strong electrostatic interactions between chitosan and nucleic acids can pose a challenge in achieving high transfection efficiency compared to other polymeric materials. To obtain higher nucleic acid delivery efficiency, chitosan-derived nanocarriers need to be developed to improve deacetylation degree, molecular weight, particle size, and N/P molar ratio [170, 171]. One possible solution to this problem is incorporating negatively charged compounds, such as γ-Polyglutamic acid (γ-PGA), into chitosan-nucleic acid complexes [172]. It was found that guanylated chitosan (GCS) promotes cellular uptake of siRNA and safely improves gene silencing efficiency. Further studies showed that the chemical coupling of salbutamol to GCS (SGCS) improved the targeting of siRNA nanoparticles to lung cells containing β2-adrenergic receptors [173]. Capel et al. modified chitosan with piperazine substitution. They showed that using modified chitosan as a carrier could mediate effective lung drug deposition after intratracheal administration and enhance siRNA-induced gene silencing in lung epithelial cells [174]. In a separate study, Jin et al. used imidazole ring-modified allantoic acid-modified chitosan as an aerosol delivery vehicle. They found that this modification resulted in higher gene transfection efficiency [175].

Klausner’s group used NOVAFECT chitosan (a currently commercially available chitosan designed by Arturrson [176]) for gene delivery studies. NOVAFECT chitosan-DNA nanoparticles injected into rat corneas showed specific expression of the luciferase gene in corneal fibroblasts and a 5.4-fold increase in expression compared to injection of polyethyleneimine-DNA nanoparticles [177]. Cheng and co-workers synthesized and applied galactosylated chitosan (GC) to encapsulate plasmids encoding macrophage colony-stimulating factor (GM-SCF) and interleukin (IL)-21. They found that following intravenous injection of GC/GM-CSF-IL-21 nanoparticles, GC/GM-CSF-IL-21 nanoparticles specifically accumulated in the liver and activated natural killer (NK) cells and cytolytic T-lymphocytes (CTLs) in tumor tissues of mice with liver metastasis model of colon cancer [178]. Similarly, Xiao et al. synthesized galactosylated chitosan-hydroxypropyltrimethylammonium (gal-HTCC) with galactosylated and quaternised chitosan. In vitro gene transfection results showed that gal-HTCC delivered pGL3 luciferase plasmid targeting to human hepatocellular carcinomas (HepG2) with remarkably higher transfection efficiencies (7–32-fold) compared with chitosan and gal-chitosan [179].

#### Poly-β-amino-ester (PBAE)

Due to the inherent cytotoxicity of PEI, there is a growing focus on developing polymers that retain the advantages of PEI but are easily degradable. One such cationic polymer is Poly-β-amino-ester (PBAE) (Fig. 4a), which has gained attention recently for its potential in the pulmonary delivery of nucleic acids. PBAE exhibits a highly adaptable chemical structure and is readily biodegradable. It can be synthesized by the Michael addition reaction [180]. The first attempt to use PBAE as a nucleic acid delivery vector was reported by Langer's team. They assembled pDNA with PBAE and found that the resulting complexes possessed a preferred nano-size and low cytotoxicity [181]. Later, Daniel G. Anderson and Robert Langer’s team explored the relationship between the capacity of pDNA-PBAE to overcome cellular barriers and the physicochemical properties of the materials [182]. They observed that the branched structure of PBAE was essential for enhancing the efficiency of nucleic acid delivery [180]. Concretely speaking, branched polymers demonstrated high transfection efficiency due to their three-dimensional (3D) spatial structure with multiple end groups [183].

The transfection efficiency of PBAE was also affected by the form of the end groups, such as end oligopeptides [184]. Anderson's group designed and synthesized a hyperbranched PBAE polymer, hDD90-118, that efficiently delivers nucleic acids to lungs by nebulization. Specifically, they delivered mRNA encoding luciferase using hDD90-118 and observed an even distribution of nanoparticles in all lung lobes. In the Ai14 reporter mouse model, they observed efficient transfection of lung epithelial cells [114] (Fig. 4b). Santangelo's group further investigated the use of hDD90-118 in nebulized delivery of nucleic acids to the lung. hDD90-118 was employed to deliver mRNA encoding virus-specific CRISPR-Cas13a protein or membrane-anchored neutralizing antibody [119, 124]. In one study, they used hDD90-118 to deliver mRNA encoding Cas13a to the respiratory tracts of mice and hamsters by nebulization, which resulted in efficient virus degradation and attenuation of respiratory infections [124]. In another experiment, the delivered cargo was exchanged for mRNA encoding a membrane-anchored neutralizing antibody. This led to the efficient expression of antibodies that alleviated the infection in the lungs of the experimental animals [119]. Afterward, they designed 166 new hyperbranched PBAE or Poly-β-amino-thio-ester (hPBATE) polymers using hDD90-118 as a precursor. Among these polymers, they found that at least five polymers, including P76, outperformed hDD90-118 in mediating lung mRNA expression. In therapeutic models of viral infections in hamsters, ferrets, cows, and non-human primates, P76 showed improved delivery efficiency in the pulmonary delivery of nucleic acids [123].

Generally, most nebulized or other inhalation methods target lung epithelial cells. Targeting pulmonary vascular endothelial cells also holds great potential in treating acute and chronic lung diseases, such as pulmonary hypertension and alveolar capillary dysplasia. In recent years, Anderson’s team has successfully achieved targeted delivery of mRNA to lung endothelial cells by combining PBAE with lipids in the form of nanoparticles (narrated in the corresponding section) [185, 186]. Modification of PBAE using lysine/histidine oligopeptides is also a promising approach. Dosta et al. found that high levels of gene silencing were observed in the pulmonary vascular endothelium after intravenous injection of PBAE-siRNA nanocomplexes, significantly reducing featureless delivery to organs such as the liver [187]. Kalinichenko’s group successfully achieved endothelial targeting after intravenous injection by unique structural design (altering the ratio of the two alkyl chains in the backbone and PEGylation and fluoride modification) of PBAE. They found that this modified PBAE could efficiently deliver pDNA to lung microcapillaries following intravenous administration [154] (Fig. 4c).

Kim et al. found that the structure of PBAE affects the biodistribution of nano complexes and gene transfection efficiency after intravenous injection. Also, they discovered by high-throughput barcode screening that PBAE NPs accumulated in the liver and spleen within 30 min of administration [188]. To target APCs in the spleen, Fornaguera et al. used oligopeptide-terminated modified PBAEs (OM-PBAEs) to deliver mRNA. They found that this newly designed material was able to accumulate efficiently in the spleen and was hardly affected by freeze-drying [189]. In a similar approach, Palmiero et al. utilized a strategy based on the synthesis of copolymers using polycaprolactone (PCL) and poly (beta-amino ester) (PBAE) through ring-opening and Michael addition polymerizations (PCL-based PBAE). They discovered that the selected ternary polymer exhibited significantly higher transfection efficiency compared to polyethyleneimine (PEI). The developed polymer primarily accumulated in the spleen with improved biocompatibility [190]. Zamboni et al. found that a primary formulation (Polymer 536, polymer to DNA weight ratio of 25) effectively transfected human Hepatocellular carcinoma (HCC) cell lines with a superior transfection efficiency over PEI and other commercially available transfection reagents (Lipofectamine™ 2000 and jetPRIME™). Notably, this biodegradable, end-modified PBAE gene delivery vector was non-cytotoxic [191]. Vaughan et al. administered PBAE nanoparticles via hepatic artery injection in a rat model of liver tumors. They found that arterial injection of PBAEs increased targeted transfection of HCC tumors compared to intravenous administration, making it a potential alternative method to Transcatheter Arterial Chemoembolization (TACE) [192]. Green's team used PBAE to deliver transgenes intracranially to enable specific gene expression in mouse gliomas. They found that the nanoparticles didn’t lose their potency after two years of freeze-drying and storage [193]. In another study, Mastorakos et al. showed that PEGylation PBAE nanoparticles delivering DNA rapidly penetrated healthy brain parenchyma and orthotopic brain tumor tissues. The nanoparticles significantly improved the survival in two aggressive orthotopic brain tumor models in rats [194].

### Lipid-based delivery system

#### Cationic lipids

**Fig. 5:**
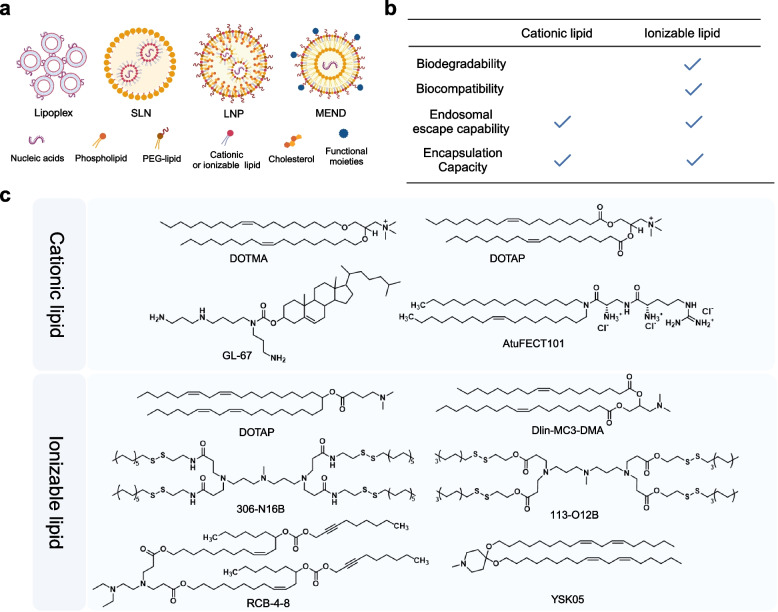
**a** Schematic illustration and **c** chemical structures of several lipids used as nucleic acids nanocarriers. **b** Comparison of the properties of cationic and ionizable lipids. Created with BioRender.com

Cationic lipids have a permanent positive charge due to the covalent binding of a positively charged head group (such as a quaternary ammonium salt, an amine, a guanidine, or a heterocyclic compound) to a hydrophobic tail group through linkages chains [195, 196] (Fig. 5). In 1987, Felgner et al. synthesized cationic lipid 1,2-di-O-octadecenyl-3-trimethylpropylammonium (DOTMA) and 1,2-Dioleoyl-sn-glycero-3-phosphoethanolamine (DOPE), which exhibited superior DNA loading capacity and efficient gene expression in vitro. It marks a pivotal moment for the application of cationic lipids in the field of nucleic acid delivery [197]. Since then, numerous research groups have embarked on employing cationic lipids as nucleic acid delivery vectors. It has been demonstrated that after intravenous injection, lipoplexes, positively charged complexes formed by cationic lipids and siRNA, can electrostatically interact with negatively charged erythrocytes to form agglomerates [198]. These agglomerates facilitate the adsorption of lipoplexes in highly dilated pulmonary capillaries, resulting in 60–70% drug accumulation in the lung within a few seconds after intravenous administration [199]. In a clinical trial, the successful treatment of patients with non-small cell lung cancer (NSCLC) was achieved through the delivery of pDNA encoding the tumor suppressor gene TUSC2/FUS1 using liposomes consisting of DOTAP and cholesterol [200]. In one study, positively charged ichthyoglobulin was used to form a complex with mRNA, and the resulting complex was further encapsulated with 1,2-dioleoyl-3-trimethylammonium propane (DOTAP). The prepared nanopcomplex induced robust cellular immune responses and delayed tumor growth [201]. High-density and low-molecular-weight PEGs have been applied to shield the surface charge of cationic lipids [202, 203]. The mucus barrier is the primary obstacle for nanoparticles delivering nucleic acids to the lung via inhalation. Taratula et al. achieved effective cell death induction and target gene silencing by co-delivering siRNA and chemotherapeutic drugs in the lung through nebulized inhalation using nanoparticles prepared with PEG-coated cationic lipid DOTAP. Also, with LHRH peptide modification of nanoparticles, they found that the drug was mainly enriched in lung tumors [204].

Cationic lipid-modified aminoglycoside (CLA) was explored to deliver mRNA to the liver. The results showed that a typical CLA named GT-EP10, delivering luciferase mRNA to mice at 0.05 mg/kg, achieved an average luminescence intensity of 10^7^ in the liver [205]. Woitok et al. produced clinically functional LNP that contained the cationic amino-lipid KL52 using a T-junction. This LNP delivering c-Jun N-terminal kinase-2 (Jnk2) siRNA achieved efficient accumulation in the liver, and Jnk2 silencing ultimately reduced carcinogenesis in a model of advanced hepatocellular carcinoma [206]. Hattori et al. developed a novel method for siRNA transfer to the liver via intravenous injection of an anionic polymer and a cationic liposome/cholesterol-modified siRNA complex (cationic liposome). They found that siRNA accumulation shifted from the lungs to the liver when injected with poly L-glutamic acid (PGA) or chondroitin C (CS) sulfate [207]. Hsu and co-workers developed cationic nanoparticles, LNP-DP1, to deliver miR-122 for restoration of dysregulated gene expression in hepatocellular carcinoma (HCC) cells [208].

Afterward, novel cationic lipids with superior transfection efficiency were developed. Commercially available cationic lipids, such as Lipofectamine 2000, have been widely used for nucleic acid delivery owing to their superior encapsulation rate and transfection effect. For example, intratracheal injection of Lipofectamine 2000 delivering PAI-1 siRNA inhibited fibroblast proliferation and promoted apoptosis of fibroblasts in a rat model of bleomycin (BLM)-induced pulmonary fibrosis [209]. Johler and colleagues found that nebulized Lipofectamine 2000 nanoparticles encapsulating EGFP mRNA achieved a transfection rate of 38% in 16HBE cells, while polymer-based nanoparticles achieved a transfection rate of only 3% [210]. Another study found that intratracheal delivery of Rip2 siRNA using Lipofectamine 2000 suppressed indicators of cigarette smoke (CS)-induced inflammation and oxidative damage, as well as inhibiting the accumulation and transcriptional activation of nuclear p65 in lung tissues [211]. Mo et al. first reported that delivery of siRNA to Huh7.5 and H4IIE hepatocellular carcinoma cells using Lipofectamine 2000 increased autophagosome levels [212]. It should be noted that multivalent cationic lipids, such as Lipofectamine®, are more toxic than monovalent cationic lipids, such as DOTAP [213].

Another notable cationic lipid is GL67, considered the “gold standard” for respiratory non-viral gene delivery vectors. GL67 was first synthesized by LEE and co-workers as a cationic lipid with spermine as the head group, conjugated to cholesterol in a T-shape structure. GL67 efficiently delivered the plasmid encoding chloramphenicol acetyltransferase (CAT) to the lungs [214]. In another study, pDNA expressing the human cystic fibrosis transmembrane conductance regulator (CFTR) was delivered as an aerosol to sheep lungs using GL67A (a mixture of GL67/DOPE/DMPE-PEG5000) [215]. Recently, DACC formulation, composed of the b-L-arginyl-2,3-L-diaminopropionic acid N-palmitoyl-N-oleyl-amide trihydrochloride (AtuFECT01), cholesterol, mPEG2000-DSPE, facilitated efficient delivery of siRNA to lung tissues and reduced VE-calmodulin mRNA expression in the lungs by approximately 50% [216]. Moreover, gene expression can be silenced in pulmonary endothelial cells by a single-dose of DACC lipid complexes [217].

#### Ionizable lipids

Recently, ionizable lipids offer pH sensitivity to enhance nucleic acid delivery in vivo [218]. In physiological conditions with a pH of 7.4, ionizable lipids are electrically neutral and have reduced interactions with anionic cell membranes, rendering them biocompatible (Fig. 5). Moreover, ionizable lipids undergo ionization in endosomes where the pH is lower than that of the extracellular environment, resulting in a positive charge, which facilitates the escape of nanoparticles from the endosomes [219, 220]. In general, ionizable lipids are less toxic and more effective at endosomal escape than cationic lipids [221, 222]. The development of ionizable lipids began with the introduction of the first ionizable lipid, 1,2-dioleoyl-3-dimethylpropanaminium (DODAP), in which the head of a cationic lipid was replaced with an ionizable molecule [223]. Subsequently, Semple et al. formulated the first LNP consisting of DODAP, which achieved 70% encapsulation efficiency with oligonucleotides [224]. To further improve the encapsulation efficiency, the ionizable lipid 1,2-dilinoleyl-N, N-dimethyl-3-aminopropane (DLin-DMA) with a superior unsaturation degree was successfully synthesized [225]. Later, a more potent ionizable lipid 2,2-dilinoleyl-4-dimethylaminoethyl-1,3-dioxolane (DLin-KC2-DMA) was developed [226]. These previous studies have led to dilinoleylmethyl‐4‐dimethylaminobutyrate (DLin‐MC3‐DMA) with potent encapsulation efficiency and transfection capability. The head linker of DLin-MC3-DMA is an ester bond, making DLin-MC3-DMA biodegradable in vivo [227]. Onpattro®, a DLin-MC3-DMA-based nucleic acid drug, marks significant progress in the field of ionizable lipid molecules. Moreover, two mRNA vaccines (mRNA-1273 and BNT162b2) based on ionizable lipids received historic emergency use approvals, which may indicate a regulatory advantage for ionizable lipids [43, 228].

**Fig. 6:**
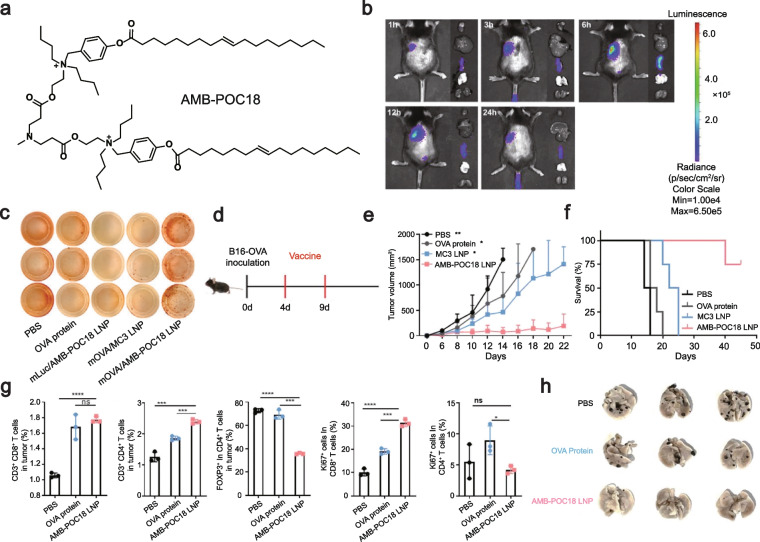
**a** The schematic of the lipid compounds (AMP-POC18) used in the article. **b** Transfection was assessed using AMB-POC18 LNPs encapsulating mRNA encoding luciferase and imaged using IVIS at different time points. **c** Detection of IFN-γ secretion using enzyme-linked immunospot analysis (ELISpot) after re-stimulation of splenocytes with SIINFEKL in vitro. **d**-**g** Antitumor effects of AMB-POC18-LNP loaded with mRNA encoding OVA as a therapeutic vaccine, evaluated by tumor volume, mouse survival, quantification of various types of immune cells, and tumor cell proliferation. **h** Lung metastases were recorded during the assessment of the therapeutic effect of vaccines on tumors by a laboratory model of tumor lung metastasis [232]. Copyright 2023, Wiley

Drew Weissman’s group synthesized a lipid library of anisodamine ligands using a one-pot, two-step modular synthesis. The best-performing ionizable lipid, AA-T3A-C12, was able to silence heat shock protein 47 by ~ 65% and was twice as effective as the MC3 LNP in a preclinical model of liver fibrosis. AA-T3A-C12/siHSP47 LNP significantly reduced collagen deposition and alleviated liver fibrosis without significant toxicity [229]. Mounting evidence has demonstrated that LNP accumulates mainly in the liver after intravenous administration [230]. Therefore, there is a great challenge to design ionizable lipids for extrahepatic delivery. Kimura et al. first discovered that LNP prepared with DODAP and DOPE under a specific ratio could selectively deliver plasmid DNA to the spleen [231]. Recently, Zhang et al. designed a novel ionizable lipid for specific delivery of mRNA to the spleen. This lipid carried a positive charge under physiological conditions. It rapidly acquired a negative charge in the presence of esterases, thus allowing stabilization of mRNA encapsulation during storage and in vivo delivery while balancing effective mRNA release from the cytoplasm [232] (Fig. 6).

To address biological barriers to nucleic acid delivery, Harashima and co-workers designed the first multifunctional envelope-type nano device (MEND) [233]. MEND is a nanoparticle that enables cell-specific targeting through surface modification by targeting ligands and incorporating pH-sensitive lipids. Pulmonary endothelial cell targeting is promising for treating various acute and chronic lung diseases [234]. Harashima’s group achieved targeted delivery to pulmonary endothelial cells by modifying a GALA peptide (developed initially as an endosomal destabilizer) on the surface of MEND [235, 236]. The same research group later designed a GALA-MEND incorporating a pH-sensitive lipid (YSK05) that efficiently delivered siRNA to pulmonary endothelial cells and inhibited lung cancer metastasis in mice. The addition of YSK05 improved the endosomal escape of MEND and enhanced the gene knock down effect in pulmonary endothelial cells [237]. Subsequently, they developed a dual-layer MEND delivery system with cell-penetrating peptide (R8 peptide). This system specifically delivered pDNA to macrophages and B cells in the spleen [238].

Xu and co-workers developed ionizable lipid 113-O12B targeting lymph nodes, which showed robust gene expression in lymph nodes. Targeted delivery of mRNA to lymph nodes increased CD8^+^ T-cell responses against a model antigen encoding ovalbumin (OVA) [239]. Most recently, the same group developed a unique LNP with a novel ionizable lipid containing an amide bond in the tail selectively delivered mRNA to the lungs. They synthesized a lipid library with amide bonds via a Michael addition reaction between the amine head and acrylamide tail and screened out the best lung-targeting lipid, 306-N16B. Interestingly, specific cell populations in the lungs can be targeted by simply switching the head structure of the LNP. In addition, through proteomics study, the researchers found that the abundant protein component in the 306-N16B corona was the fibrinogen beta chain. Then, they encapsulated mRNA encoding mouse tuberous sclerosis complex 2 (Tsc2) with 306-N16B. Following intravenous injection, the resulting nanocomplex restored the expression of the Tsc2 tumor suppressor for the treatment of lung lymphangioma (LAM) [109].

In a separate study, Daniel G. Anderson's team constructed OF-Deg-Lin lipids that induced 85% of protein expression in the spleen and effectively targeted B lymphocytes in vivo (~ 7%), showing the potential to modulate B cell function [240]. The same research team later synthesized 720 biodegradable lipids based on a three-component reaction system and screened out the top-performing ionizable lipid, RCB-4–8. In particular, the carbonate groups in RCB-4–8 rendered it more biodegradable than lipids with ester groups. They found that RCB-4–8 LNPs efficiently delivered mRNA to club cells and ciliated cells (the two significant subtypes of airway epithelial cells) via intratracheal delivery. Moreover, the pulmonary transfection efficiency of RCB-4–8 LNPs was superior over that of LNPs formulated with DLin-MC3-DMA, and the addition of the cationic lipid DOTAP further improved luciferase expression upon intratracheal administration. Also, they proposed a strategy for co-delivery of SpCas9 with adeno-associated virus (AAV) and RCB-4–8. Following intratracheal administration, immunostaining analysis of lung tissue sections showed activation of the tdTomato fluorescent reporter gene in 17.0 ± 5.0% of pulmonary cells [141].

#### Other lipids

In some studies, solid lipid nanoparticles (SLNs) were also employed for the pulmonary delivery of nucleic acids. Jacobson et al. demonstrated that siRNA-DOTAP complexes can be efficiently encapsulated within the neutral hydrophobic cores of SLNs using a hydrophobic ion-pair approach [241]. Recently, Wang and co-workers successfully delivered tumor necrosis factor-α (TNF-α) siRNA-SLNs by dry powder inhalation through a simulated mucus layer and achieved gene silencing in pulmonary macrophages and epithelial cells [242]. Most recently, Siegwart's group developed selective organ targeting (SORT) technology, which modulates the molar composition and internal charge of LNPs by adding new lipid molecules to conventional four-component LNPs. The addition of the SORT molecule to the original four-component LNP formulation allows for liver, lung, and spleen-specific targeting. They found that when negatively charged 1,2-dioleoyl-sn-glycero-3-phosphate (18PA) was added at 10–40%, SORT LNPs selectively accumulated and induced protein expression in the spleen, with no luciferase expression in other organs. Optimal lung delivery was achieved when DOTAP was added up to 50%, and complete liver targeting was reached by adding 20% DODAP. Based on SORT, ReCode has developed an inhalable mRNA vaccine (RCT1100) against Primary Ciliary Dyskinesia (PCD) that has entered Phase I clinical studies. The vaccine showed high DNAI1 protein expression, rapid LNP clearance in ciliated, rod, and basal cells, excellent tolerability, and the potential for repeated administration [147]. The same group later synthesized a novel LNP delivery system called iPLNPs, which consist of novel phospholipids (iPhos) with endosomal escape properties. They found that adding DDAB to the top-performing iPhos 9A1P9 effectively mediated enhanced mRNA delivery and CRISPR-Cas9 gene editing in the lungs [243].

### Polymer–lipid hybrid delivery system

Polymer–lipid hybrid nanoparticles may improve the safety and long-lasting efficacy of single nanomaterial for nucleic acid delivery by taking advantage of the complementary properties of polymer and lipid nanoparticles [151, 154, 187, 188]. Thanki and colleagues developed a lipid-polymer hybrid nanoparticle by incorporating poly(DL-lactic-co-glycolic acid) (PLGA) with DOTAP for siRNA delivery. Compared to DOTAP alone, this hybrid nanoparticle effectively increased the siRNA payload and greatly optimized the gene-silencing effect [244, 245]. Meyer et al. designed self-assembled lipid/polymer hybrid (LPH) nanoparticles of PLGA with DOTAP or MC3. It was demonstrated that LPH nanoparticles accumulated predominantly in the liver post intravenous administration, whereas luciferase proteins were specifically expressed in the spleen and lungs [246]. Matsumoto et al. designed PEI lipopolyplexes with DOTMA and pDNA. The charge ratios of the complexes to pDNA were calculated from the molar values of the nitrogen of PEI and the nitrogen of DOTMA to pDNA phosphate. As the charge ratio was four, the lipopolyplexes selectively expressed the gene in the spleen [247].

Recently, Philip J. Santangelo's group developed a novel polymer–lipid hybrid nanoparticle named NLD1 using DOTAP in conjunction with 7C1 (a low molecular weight PEI) [248]. Nebulized NLD1 mRNA encoding broadly neutralizing antibodies targeting haemagglutinin substantially prevented lethal H1N1 influenza virus infection in mice, with higher delivery efficiency compared to MC3 and cKK-E12 [249]. Anderson's research team demonstrated that intravenous injection of PBAE delivering mRNA remarkably accumulated in the lungs of mice [186]. Moreover, they found that adding PEG-lipid to PBAE reduced the nanoparticle size and further increased its specificity towards the lungs [185]. The resulting nanoparticles efficiently delivered Cre mRNA to pulmonary endothelial cells and immune cells. Recently, the same group optimized the properties of PBAE by incorporating a third hydrophobic alkylamine monomer to form a terpolymer. This copolymer, D90-C12-103, achieved DNA transfection with 1–2 orders of magnitude higher efficacy than other transfection reagents such as C12-200 and PEI [250] (Table 2).

**Table 2:** List of nanomaterials for tissues-targeted delivery of nucleic acids

Nanocarrier	Composition	Cargo	Targeted cell types or treated diseases	Administration route	Ref
Hybrid nanoparticles	PLGA-PEG/G0-C14	siRNA	Idiopathic pulmonary fibrosis (IPF)	Inhalation	[79]
Polymer	Fluorinated PEI	siRNA	Liver disease	Intravenous injection	[103]
Polymers	PEI-PEG	siRNA	Bronchial and alveolar cells	Intratracheal	[104]
Ionizable lipids	306-N16B/Cholesterol /DOPC or DOPE or DSPC/mPEG2000-DMG	mRNA	Pulmonary lymphangioleiomyomatosis	Intravenous injection	[109]
Polymers	Hyperbranched-PBAE (hDD90-118)	mRNA	Pulmonary epithelial cells	Inhalation	[114]
Polymers	Hyperbranched-PBAE (hDD90-118)	mRNA	Virus infection	Inhalation	[119]
Polymers	PBATE (P76)	mRNA	Virus infection	Inhalation	[123]
Polymers	PEI	pDNA	Mucosal epithelial cells	Intranasal	[150]
Polymers	PEI-PEG	siRNA	Lung cells (leucocytes)	Intratracheal	[157]
Polymers	PEI	siRNA	Bronchial and alveolar cells	Intratracheal	[158, 159]
Polymers	PBAE	pDNA	Hepatocellular carcinoma	Intratumoral injection	[191]
Polymers	PBAE (P22-F1) (Fluorinated)	pDNA	Pulmonary endothelial cells	Intravenous	[154]
Polymers	PEI/DOPS	pDNA	Spleen	Intravenous injection	[161]
Polymers	Chitosan	siRNA	Lung cancer	Intratracheal	[168]
Polymers	Chitosan (Piperazine-substituted)	siRNA	Lung cancer	Intratracheal	[174]
Polymers	Chitosan	pDNA	Liver	Intravenous injection	[178]
Hybrid nanoparticles	Cholesterol/PBAE/PEG2000 PE/DOPE	mRNA	Pulmonary endothelium and immune cells	Intravenous injection	[185]
Polymers	PBAE (C6-KH) (Lysine-/histidine-oligopeptide modified)	siRNA	Pulmonary endothelial cells	Intravenous	[187]
Polymers	PBAE	pDNA	Glioblastoma	Intracranial administration	[189]
Polymers	PBAE	pDNA	Malignant Brain Tumors	Convection-enhanced delivery	[190]
Cationic lipids	DOTAP/Cholesterol	pDNA	Lung cancer	Intravenous injection	[200]
Cationic lipids	DOTAP/Cholesterol/DSPE-PEG-2000	mRNA	Lung cancer	Intranasal	[201]
Cationic lipids	DOTAP/DSPE-PEG/LHRH peptide	siRNA	Lung cancer	Inhalation	[204]
Cationic lipids	Lipofectamine 2000 transfection agent	mRNA	Pulmonary epithelial cells	Nebulization	[210]
Cationic lipids	Lipofectamine 2000 transfection agent	siRNA	Chronic obstructive pulmonary disease (COPD)	Intratracheal	[211]
Cationic lipids	Lipofectamine 2000 transfection agent	siRNA	Hepatoma Cells	NA	[212]
Cationic lipids	GL67/DOPE	pDNA	Cystic Fibrosis	Intranasal	[214]
Cationic lipids	GL67/DOPE/DMPE/ PEG5000	pDNA	Cystic fibrosis	Inhalation	[215]
Cationic lipids	AtuFECT01/DPhyPE/DSPE-PEG	siRNA	Pulmonary endothelial cells	Intravenous injection	[216]
Cationic lipids	AtuFECT01/Cholesterol/mPEG2000-DSPE	siRNA	Pulmonary endothelial cells	Intravenous injection	[217]
Ionizable lipids	AA-T3A-C12/Cholesterol/DSPC/C14-PEG 2000	siRNA	Liver fibrosis	Intravenous injection	[229]
Ionizable lipids	DODAP/Cholesterol/DOPE/DMG-PEG 2000	pDNA	Spleen	Intravenous injection	[231]
Ionizable lipids	AMB-POC18/DOPE/PEG-DMG 2000	mRNA	Spleen	Intravenous injection	[232]
MEND	EPC/Cholesterol/STR-mPEG/Chol-GALA	siRNA	Pulmonary endothelium cells	Intravenous injection	[236]
MEND	YSK05/Cholesterol/EPC/DMG-PEG/Chol-GALA	siRNA	Pulmonary endothelial cells	Intravenous injection	[237]
MEND	DOPE/STR-R8/YSK05/Cholesterol/DMG-PEG	pDNA	Spleen B cells	Intravenous injection	[238]
Ionizable lipids	113-O12B/Cholesterol /DSPC/PEG2000-DMG	mRNA	Lymph node	Subcutaneous injection	[239]
Ionizable lipids	OF-Deg-Lin/Cholesterol/DOPE/C14-PEG 2000	mRNA	Spleen B lymphocytes	Intravenous injection	[240]
SLNs	DOTAP/Lecithin/Cholesterol/PHC	siRNA	Alveolar macrophages or pulmonary epithelial cells	Dry powder inhalation	[242]
Hybrid nanoparticles	PLGA/DSPC/DMG-PEG/DOTAP or MC3	mRNA	Spleen	Intravenous injection	[246]
Hybrid nanoparticles	7C1/Cholesterol/C14-PEG2000/DOTAP	mRNA	H1N1 influenza virus infection	Nebulization	[249]

## Outlook and conclusion

Initially, viral vectors were utilized for nucleic acid delivery. However, their potential immunogenicity and safety concerns have posed insurmountable challenges, leading to a gradual shift towards synthetic nanocarriers. Recently, polymers have been one of the pioneering nanomaterials employed for the delivery of nucleic acids. Although polymers possess the advantages of easy synthesis and variable chemical structures, they often face restrictions in terms of biodegradability [251, 252]. Undoubtedly, the most successful nucleic acid carriers in the current stage are LNPs. A growing body of studies has confirmed that LNPs have been the most advanced nucleic acid delivery systems to date. They have gained extensive usage, including two approved mRNA vaccines on the market against SARS-CoV-2. Nevertheless, conventional LNPs are difficult to specifically target extrahepatic organs [55]. So far, the focus has shifted to designing delivery systems that can effectively deliver therapeutic agents to targeted tissues and cells [253]. Researchers have found that the tissue selectivity of nanoparticles is closely related to factors such as the surface charge of lipid fractions. Although cationic lipid-based LNPs consisting of cations, such as DOTAP, have achieved pulmonary delivery of nucleic acids to a certain extent, there are still some potential safety hazards associated with the unavoidable cytotoxicity of positively charged substances in vivo [251]. The emergence of ionizable lipids, which carry a positive charge only in a specific physiological environment, avoids the toxicity of cationic lipids and brings superior transfection efficiency [38, 222, 254–258]. Currently, most lung-targeted nucleic acid products that have entered the clinical research stage have been developed based on ionizable lipids, including MRT5005® RCT1100®. Recent studies have explored new approaches, such as attaching peptides or antibodies on the surface of nanoparticles, to achieve active targeting. The researchers have investigated the properties of materials that influence delivery efficacy and intend to provide solutions to overcome the limitations of existing materials [259].

We must acknowledge that existing technologies still cannot solve all problems, partly due to the challenges in material synthesis and design, and partly due to the factors determining the targeted effects of materials not being fully elucidated [260]. The key to developing next-generation nucleic acid delivery vectors with optimal properties lies on overcoming complex physiological barriers. Some strategies have been applied to overcome physiological barriers, such as the blood–brain barrier (BBB) [261]. The characteristics of nanoparticles, such as particle size and surface charge, play vital roles in overcoming the physiological barriers. When developing innovative nanomaterials, it is crucial to comprehensively assess their characteristics, including cytotoxicity, nucleic acid loading capacity, endosomal escape efficiency, and storage stability. A thorough exploration of the relationship between material properties and tissue targeting ability should be conducted. Most recently, high-throughput screening methods, such as DNA barcoding technology, have been employed to explore the active targeting ability of nanomaterials [262, 263]. The past decade has witnessed the accelerating development of nanomaterials for nucleic acid delivery. We envision that with the rapid development of biomaterial science, nucleic acid drugs will play central roles in the prevention and treatment of various diseases in the near future.

## Data Availability

Not applicable.
